# PD-1/PD-L1 checkpoint inhibitors during late stages of life: an ad-hoc analysis from a large multicenter cohort

**DOI:** 10.1186/s12967-021-02937-9

**Published:** 2021-06-24

**Authors:** Daniele Santini, Tea Zeppola, Marco Russano, Fabrizio Citarella, Cecilia Anesi, Sebastiano Buti, Marco Tucci, Alessandro Russo, Maria Chiara Sergi, Vincenzo Adamo, Luigia S. Stucci, Melissa Bersanelli, Giulia Mazzaschi, Francesco Spagnolo, Francesca Rastelli, Francesca Chiara Giorgi, Raffaele Giusti, Marco Filetti, Paolo Marchetti, Andrea Botticelli, Alain Gelibter, Marco Siringo, Marco Ferrari, Riccardo Marconcini, Maria Giuseppa Vitale, Linda Nicolardi, Rita Chiari, Michele Ghidini, Olga Nigro, Francesco Grossi, Michele De Tursi, Pietro Di Marino, Laura Pala, Paola Queirolo, Sergio Bracarda, Serena Macrini, Stefania Gori, Alessandro Inno, Federica Zoratto, Enrica T. Tanda, Domenico Mallardo, Maria Grazia Vitale, Thomas Talbot, Paolo A. Ascierto, David J. Pinato, Corrado Ficorella, Giampiero Porzio, Alessio Cortellini

**Affiliations:** 1 Medical Oncology, Campus Bio-Medico University, Rome, Italy; 2 Medical Oncology Unit, University Hospital of Parma, Parma, Italy; 3 Medical Oncology Unit, Department of Biomedical Sciences and Human Oncology, University of Bari, Bari, Italy; 4 National Cancer Research Center, Tumori Institute IRCCS Giovanni PaoloII, Bari, Italy; 5 Medical Oncology, A.O. Papardo & Department of Human Pathology, University of Messina, Messina, Italy; 6 Department of Medicine and Surgery, University of Parma, Parma, Italy; 7 IRCCS Ospedale Policlinico San Martino, Genova, Italy; 8 UOC Oncologia Ascoli Piceno – San Benedetto del Tronto, Area Vasta 5, ASUR Marche, Ancona, Italy; 9 Medical Oncology Unit, Sant’ Andrea Hospital of Rome, Rome, Italy; 10 Department of Clinical and Molecular Medicine, Sapienza University of Rome, Rome, Italy; 11 Medical Oncology (B), Policlinico Umberto I, “Sapienza” University of Rome, Rome, Italy; 12 Medical Oncology, Azienda Ospedaliero-Universitaria Pisana, Pisa, Italy; 13 Medical Oncology, University Hospital of Modena, Modena, Italy; 14 UOC Oncologia Padova Sud - AULSS6 Euganea, Padova, Italy; 15 Medical Oncology Unit, Fondazione IRCCS Ca Granda Ospedale Maggiore Policlinico, Milan, Italy; 16 Medical Oncology, ASST Sette Laghi, Ospedale di Circolo e Fondazione Macchi, Varese, Italy; 17 Division of Medical Oncology, University of Insubria, Varese, Italy; 18 Department of Medical, Oral & Biotechnological Sciences, University G. D’Annunzio, Chieti-Pescara, Italy; 19 Clinical Oncology Unit, S.S. Annunziata Hospital, Chieti, Italy; 20 Division of Medical Oncology for Melanoma, Sarcoma, and Rare Tumors, IEO, European Institute of Oncology IRCCS, Milan, Italy; 21 S.C. Medical Oncology, Azienda Ospedaliera S. Maria, Terni, Italy; 22 Oncology Unit, IRCCS Ospedale Sacro Cuore Don Calabria, Negrar, VR Italy; 23 Medical Oncology, Santa Maria Goretti Hospital, Latina, Italy; 24 Genetics of Rare Cancers, Department of Internal Medicine and Medical Specialties, University of Genoa, Genoa, Italy; 25 Melanoma, Cancer Immunotherapy and Development Therapeutics Unit, Istituto Nazionale Tumori-IRCCS Fondazione “G. Pascale”, Naples, Italy; 26 Department of Surgery and Cancer, Faculty of Medicine, Imperial College London, Hammersmith Hospital, Du Cane Road, London, W12 0HS UK; 27 Division of Oncology, Department of Translational Medicine, University of Piemonte Orientale, Novara, Italy; 28 Medical Oncology Unit, St. Salvatore Hospital, L’Aquila, Italy; 29 Department of Biotechnological and Applied Clinical Sciences, University of L’Aquila, L’Aquila, Italy

**Keywords:** Immunotherapy, Immune checkpoint inhibitors, End-of-life, Palliative care, Appropriateness

## Abstract

**Background:**

The favourable safety profile and the increasing confidence with immune checkpoint inhibitors (ICIs) might have boosted their prescription in frail patients with short life expectancies, who usually are not treated with standard chemotherapy.

**Methods:**

The present analysis aims to describe clinicians’ attitudes towards ICIs administration during late stages of life within a multicenter cohort of advanced cancer patients treated with single agent PD-1/PD-L1 checkpoint inhibitors in Italy.

**Results:**

Overall, 1149 patients with advanced cancer who received single agent PD-1/PD-L1 checkpoint inhibitors were screened. The final study population consisted of 567 deceased patients. 166 patients (29.3%) had received ICIs within 30 days of death; among them there was a significantly higher proportion of patients with ECOG-PS ≥ 2 (28.3% vs 11.5%, p < 0.0001) and with a higher burden of disease (69.3% vs 59.4%, p = 0.0266). In total, 35 patients (6.2%) started ICIs within 30 days of death; among them there was a higher proportion of patients with ECOG-PS ≥ 2 (45.7% vs 14.5%, p < 0.0001) and with a higher burden of disease (82.9% vs 60.9%, p = 0.0266). Primary tumors were significantly different across subgroups (p = 0.0172), with a higher prevalence of NSCLC patients (80% vs 60.9%) among those who started ICIs within 30 days of death. Lastly, 123 patients (21.7%) started ICIs within 3 months of death. Similarly, within this subgroup there was a higher proportion of patients with ECOG-PS ≥ 2 (29.3% vs 12.8%, p < 0.0001), with a higher burden of disease (74.0% vs 59.0%, p = 0.0025) and with NSCLC (74.0% vs 58.8%, p = 0.0236).

**Conclusion:**

Our results confirmed a trend toward an increasing ICIs prescription in frail patients, during the late stages of life. Caution should be exercised when evaluating an ICI treatment for patients with a poor PS and a high burden of disease.

**Supplementary Information:**

The online version contains supplementary material available at 10.1186/s12967-021-02937-9.

## Introduction

Several studies showed that palliative chemotherapy does not improve the quality of life (QoL) of patients with end-stage cancer and has a detrimental effect in patients with good performance status due to toxicities [1–3]. After the advent of immune checkpoint inhibitors (ICIs), the treatment paradigm of several malignancies has radically changed, and although ICIs are associated with class-specific inflammatory side effects, namely immune-related adverse events (irAEs), they are characterized by an overall favourable safety profile as compared to chemotherapy [4].

Clinician awareness of irAEs clinical presentation, diagnosis and management has increased over time. As consequence of this increasing confidence, attitudes towards ICIs prescription in frail patients, who are usually unfit for standard chemotherapy, might have increased too. This attitude has been described as "desperation oncology" [5], an unbalance between hope and reality that produces detrimental effects on the patient's QoL and might have a huge economic impact on healthcare systems [6, 7].

There is lacking literature exploring the use of ICIs during end-of-life stages, therefore, in the absence of high levels of evidence, some competitive factors could weigh on medical decisions, including reports of “miracles” found in all human-interest stories.

Against this background, we conducted this ad-hoc analysis within a large multicentre cohort of advanced cancer patients treated with single agent PD-1/PD-L1 checkpoint inhibitors in Italy.

## Materials and Methods

### Study design

The aim of the present analysis was to describe clinicians’ attitudes towards single agent PD-1/PD-L1 checkpoint inhibitors administration and prescription during late stages of life, among a multicenter cohort of advanced cancer patients treated in clinical practice in Italy [8–15] (Additional file 1, Table S1). This "ad-hoc" analysis has been performed on a cohort of patients already collected, after a follow-up update. Considering this, and the descriptive overarching aim, we did not perform a formal power calculation.

Following a request for a data update, 19 Institutions participated (Additional file 1: Table S1); consecutive patients with confirmed stage IV cancer who received immunotherapy from June 2014 to June 2020, as 1st or subsequent line were screened, data cut-off period was December 2020. Patients who died, with available information about the last administration of immunotherapy were included.

To provide a detailed picture of trends in ICIs administration during late stages of life we established the following clinical endpoints of interest:

- Having received ICIs within 30 days of death [16];
- Having started ICIs within 30 days of death [17];
- Having started ICIs within 3 months of death [18].

An explorative univariable analysis was also performed, in order to evaluate whether any baseline (at ICIs initiation) patient characteristics were associated with clinical endpoints of interests. The considered baseline features were: primary tumor types (NSCLC, melanoma, renal cell carcinoma and others), age (< 70 *vs* ≥ 70 years old/ < 60 *vs* ≥ 60 years old), gender (male *vs* female), Eastern Cooperative Oncology Group-Performance Status (ECOG-PS) (0–1 *vs* ≥ 2), burden of disease (number of metastatic sites ≤ 2 *vs* > 2), and treatment line (first *vs* second *vs* further lines). Additionally, to depict the trends towards ICIs administration during late stages of life over time, we also reported the crude incidence of the three endpoints of interest across the years, clustered as follow: 2014–2015, 2016–2017, 2018–2020.

In order to provide further insights on clinicians’ attitudes towards continuing ICIs until the late stages of life, the associations between administration of ICI within 30 days of death and best response to ICI/time to treatment failure (TTF) have been evaluated. Methods regarding response evaluation have been already reported [1–8]. Best response to ICIs was categorized as partial response (PR)/complete response (CR) *vs* stable disease (SD)/progressive disease (PD). Patients who did not undergo formal radiological assessment were excluded from this analysis. TTF was defined as the time from ICI initiation to treatment discontinuation for whatever cause and was categorized as ≥ 3 months *vs* < 3 months. Lastly, we also explored the achieved disease control rate (DCR, defined as the portion of patients experiencing PR/CR/SD) among those patients who started ICI within 3 months of death who underwent a formal radiological assessment.

Baseline patient characteristics were reported with descriptive statistics. χ2 and Fisher’s exact tests was used for all univariable analyses as appropriate. Median TTF was estimated evaluated using the Kaplan–Meier method. The alpha level for all analyses was set to p < 0.05. All statistical analyses were performed using MedCalc Statistical Software version 19.3.1 (MedCalc Software Ltd, Ostend, Belgium; https://www.medcalc.org; 2020).

## Results

**Fig. 1:**
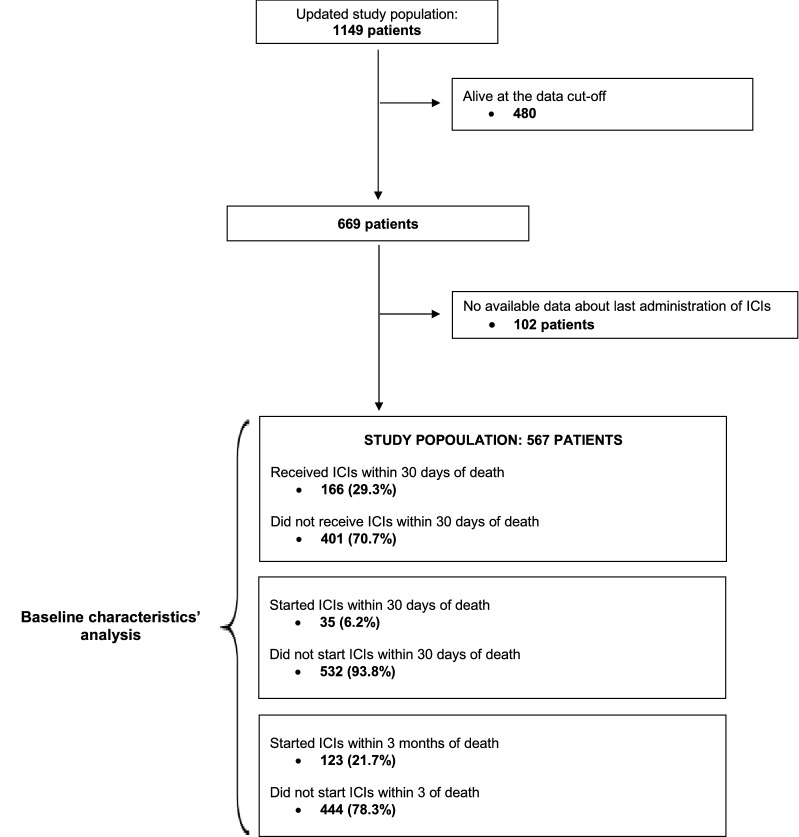
Study flow diagram

Overall, 1149 patients with advanced cancer who received single agent PD-1/PD-L1 checkpoint inhibitors were screened as part of the data update process. At data cut-off, 480 patients were alive while 102 patients had missing information about the last administration of immunotherapy. The final study population consisted of 567 deceased patients. Figure 1 reported the study flow diagram.

Table 1 summarizes baseline patient characteristics for the whole population and the subgroup analysis based on receipt of ICIs within 30 days of death. One hundred and sixty-six patients (29.3%) received ICIs within 30 days of death. Among them there was a significantly higher proportion of patients with ECOG-PS ≥ 2 (28.3% vs 11.5%, p < 0.0001) and with a higher burden of disease (69.3% vs 59.4%, p = 0.0266). No significant association was found with respect to age (p = 0.9163), gender (p = 0.1600), primary tumor (p = 0.0707) or treatment line (p = 0.8895). Administration of ICIs within 30 days of death was associated with a shorter TTF (55.4% vs 41.6%, p = 0.0028), while no association with the achieved best response was reported.

**Table 1:** Baseline characteristics of the overall study population and of patients grouped according to the receipt of ICIs within 30 days of death

	Overall	No ICIs administration within 30 days of death	ICIs administration within 30 days of death	
	N° (%)	N° (%)	N° (%)	
	567	401	166	
Age				
< 60 years old	148 (26.1)	111 (27.7)	37 (22.3)	P = 0.1839 P = 0.9163
≥ 60 years old	419 (73.9)	290 (72.3)	129 (77.7)	
< 70 years old	323 (57.0)	229 (57.1)	94 (56.6)	
≥ 70 years old	244 (43.0)	172 (42.9)	72 (43.4)	
Gender				P = 0.1600
Female	192 (33.9)	143 (37.5)	49 (29.5)	
Male	375 (66.1)	258 (64.3)	117 (70.5)	
ECOG PS				P < 0.0001
0—1	474 (83.6)	355 (88.5)	119 (71.7)	
≥ 2	93 (16.4)	46 (11.5)	47 (28.3)	
Primary tumor				P = 0.0707
NSCLC	352 (62.1)	239 (59.6)	113 (68.2)	
Melanoma	111 (19.6)	89 (22.2)	22 (13.3)	
Renal cell carcinoma	83 (14.6)	60 (15.0)	23 (13.9)	
Others	21 (3.7)	13 (3.2)	8 (4.8)	
No. of metastatic sites				P = 0.0266
≤ 2	214 (37.7)	163 (40.6)	51 (30.7)	
> 2	353 (62.3)	238 (59.4)	115 (69.3)	
Type of anti-PD-1/PD-L1 agent				P = 0.5445
Pembrolizumab	168 (29.6)	123 (30.7)	45 (27.1)	
Nivolumab	370 (65.3)	258 (64.3)	112 (67.5)	
Atezolizumab	18 (3.2)	11 (2.7)	7 (4.2)	
Others	11 (1.9)	9 (2.2)	2 (1.2)	
Treatment line				P = 0.8895
First	173 (30.5)	123 (30.7)	50 (28.9)	
Second	324 (57.1)	227 (56.6)	97 (58.4)	
Further lines	70 (12.3)	51 (12.7)	19 (11.4)	
Best response^a^				P = 0.4693
PR/CR	403 (79.5)	300 (78.7)	103 (81.7)	
PD/SD	104 (20.5)	81 (21.3)	23 (18.3)	
Time to treatment failure				P = 0.0028
≥ 3 months	308 (54.3)	234 (58.4)	74 (44.6)	
< 3 months	259 (45.7)	167 (41.6)	92 (55.4)	

^a^507 evaluable patients for best response. ICI: immune checkpoint inhibitor; ECOG-PS: eastern cooperative oncology group performance status; NSCLC: non-small cell lung cancer; PD-1/PD-L1: programmed death-1/programmed death-ligand 1; PR: partial response; CR: complete response; PD: progressive disease; SD: stable disease

Overall, 35 patients (6.2%) started ICIs within 30 days of death (Table 2); among them there was a higher proportion of patients with ECOG-PS ≥ 2 (45.7% vs 14.5%, p < 0.0001) and with a higher burden of disease (82.9% vs 60.9%, p = 0.0266). No significant association was found with respect to age (p = 0.2810), gender (p = 0.7536), or treatment line (p = 0.1822), while primary tumors were significantly different across the subgroup (p = 0.0172), with a high prevalence of NSCLC patients (80% vs 60.9%) among those who started ICIs within 30 days of death.

**Table 2:** Patients' characteristics according to ICIs initiation within 30 days of death

	Control	ICIs started within 30 days of death	
	N° (%)	N° (%)	
	532 (93.8)	35 (6.2)	
Age			
< 60 years old	142 (26.7)	6 (17.1)	P = 0.2132 P = 0.2810
≥ 60 years old	390 (73.3)	29 (82.9)	
< 70 years old	300 (56.4)	23 (65.7)	
≥ 70 years old	232 (43.6)	12 (34.3)	
Gender			P = 0.7536
Female	181 (34.0)	11 (31.4)	
Male	351 (66.0)	24 (68.6)	
ECOG PS			P < 0.0001
0—1	455 (85.5)	19 (54.3)	
≥ 2	77 (14.5)	16 (45.7)	
Primary tumor			P = 0.0172
NSCLC	324 (60.9)	28 (80)	
Melanoma	108 (20.3)	3 (8.6)	
Renal cell carcinoma	82 (15.4)	1 (2.9)	
Others	18 (3.4)	3 (8.6)	
No. of metastatic sites			P = 0.0095
≤ 2	208 (39.1)	6 (17.1)	
> 2	324 (60.9)	29 (82.9)	
Treatment line			P = 0.1822
First	158 (29.7)	15 (42.9)	
Second	306 (57.5)	18 (51.4)	
Further lines	68 (12.8)	2 (5.7)	

ICI: immune checkpoint inhibitor; ECOG-PS: eastern cooperative oncology group-performance status; NSCLC: non-small cell lung cancer

In total, 123 patients (21.7%) started ICIs within 3 months of death (Table 3). Similarly, within this subgroup there was a higher proportion of patients with ECOG-PS ≥ 2 (29.3% vs 12.8%, p < 0.0001), with a higher burden of disease (74.0% vs 59.0%, p = 0.0025) and with NSCLC (74.0% vs 58.8%, p = 0.0236). Treatment line distribution was also significantly different between patients who and who did not started ICIs within 3 months of death (p = 0.0189), while no association was reported regarding gender (p = 0.3171) and age (p = 0.8261). Among the 80 evaluable patients who started ICIs within 3 months of death, the DCR was 3.7% (95%CI: 0.77–10.9), while among the 427 who did not start ICIs within 3 months of death DCR was 54.1% (95%CI: 47.3–61.5) (p < 0.0001).

**Table 3:** Patients' characteristics according to ICIs initiation within 3 months of death

	Control	ICIs started within 3 months of death	
	N° (%)	N° (%)	
	444 (78.3)	123 (21.7)	
Age			
< 60 years old	124 (27.9)	24 (19.5)	P = 0.0603 P = 0.8261
≥ 60 years old	320 (72.1)	99 (80.5)	
< 70 years old	254 (57.2)	69 (56.1)	
≥ 70 years old	190 (42.8)	54 (43.9)	
Gender			P = 0.3171
Female	155 (34.9)	37 (30.1)	
Male	289 (65.1)	86 (69.9)	
ECOG PS			P < 0.0001
0—1	387 (87.2)	87 (70.7)	
≥ 2	57 (12.8)	36 (29.3)	
Primary tumor			P = 0.0236
NSCLC	261 (58.8)	91 (74.0)	
Melanoma	94 (21.2)	17 (13.8)	
Renal cell carcinoma	71 (16.0)	12 (9.8)	
Others	18 (4.1)	3 (2.4)	
No. of metastatic sites			P = 0.0025
≤ 2	182 (41.0)	32 (26.0)	
> 2	262 (59.0)	91 (74.0)	
Treatment line			P = 0.0189
First	138 (31.1)	35 (28.5)	
Second	243 (54.7)	81 (65.9)	
Further lines	63 (14.2)	7 (5.7)	

ICI: immune checkpoint inhibitor; ECOG-PS: eastern cooperative oncology group-performance status; NSCLC: non-small cell lung cancer

**Fig. 2:**
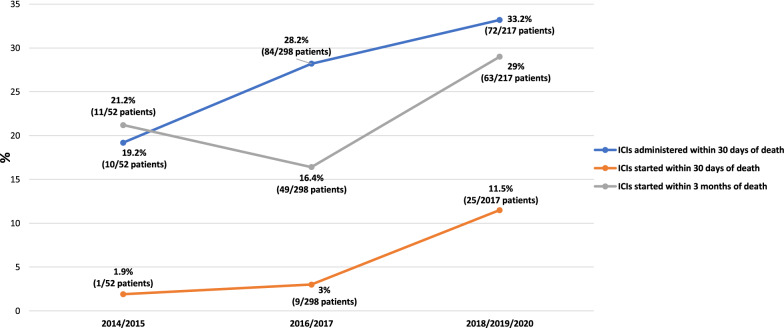
Trends towards ICIs administration during late stages of life

Figure 2 reports the analysis of the three endpoints of interest over time, clearly revealing an increasing trend of ICIs administration within 30 days and ICIs initiation within 30 days/3 months of death, over the years.

## Discussion

An increasing tendency towards ICIs prescription and administration during end-of-life stages has been already reported. A multicenter analysis of advanced urothelial carcinoma patients reported an increase of ICIs administration within 60 days of death, from 1 to 23% between the final quarter of 2015 and 2017, respectively [19]. Similarly, Glisch and colleagues reported that patients receiving ICIs within 30 days of death have a poorer PS, do not receive subsequent anticancer treatments and are more likely to die in hospital rather than in hospice [20]. Analogous results have also been demonstrated among NSCLC patients [21], while Durbin et al. specifically described a cohort of patients initiating ICIs in the inpatient setting, confirming their poor overall outcome [22].

Our study confirmed that in clinical practice, a not negligible portion of patients received immunotherapy with single agent PD-1/PD-L1 inhibitors within 30 days of death, as well as started ICIs within 3 months/30 days of death. Overall, these patients had a high burden of disease, presented features of frailty such as poorer PS, and in the subgroup of patients initiating ICI within 3 months of death were often being treated with an advanced line of therapy. We also found that those who started ICIs within 3 months and 30 days of death were more likely to be NSCLC patients, mirroring what have been already reported by Durbin et al. [22]. Even this finding reflects a feature of frailty, as NSCLC is known to be a more aggressive disease as compared to melanoma and renal cell carcinoma. Unsurprisingly, the achieved clinical outcomes for patients who initiated ICIs within 3 months of death were disappointing with a DCR of 3.7%.

Interestingly, the attitude of administering ICIs during the late stages of life was not associated with patient age in our cohort, supporting that age does not impair ICI efficacy [23, 24], while decision to continue ICIs until the late stages of life did not depend on previous clinical benefit, confirming that the choice to extend ICIs therapy beyond a clinically useful time window is (often) founded by a "desperation" approach, rather than guided by evidence of previous clinical benefit.

Our exploratory analysis of ICI prescribing trends over time, clearly confirmed that there is a recent increasing tendency for clinicians to trial ICI therapy in very advanced cancer patients, likely depicting increasing confidence in prescribing ICIs for frail patients, during the late stages of life, relative to that reported with standard chemotherapy [16, 17]. These findings, if on one hand reflect the better safety profile of ICIs, which allows their administration in patients unfit for chemotherapy, on the other hand must make us pause for thought. Though a proper life expectancy estimation might be challenging and inaccurate, we clearly revealed that caution should be exercised when considering ICI treatment for patients (especially NSCLC) with a poor PS, a high burden of disease, and in an advance line of treatment. These hallmarks of frailty might represent a wake-up call for clinicians, to take into consideration to ensure appropriateness of ICI prescription.

However, our study reports a snapshot of the Italian clinical practice, which has its own peculiarities. The National Health System in Italy is "universalistic", meaning that it is entirely government-funded and guarantees costly therapies and procedures to all oncological patients, regardless of their income or insurance status. Although this approach protects the welfare of all citizens, ensuring free access to care and services equitably, other healthcare systems with mixed coverage schemes (e.g. with private health insurances), might be more efficient in monitoring and analyzing the cost/benefit ratio of anticancer treatments, as clearly reported by Glisch C et al. and Durbin SM et al. [20–22]. Nonetheless, the Italian drug regulatory agency, namely AIFA (Agenzia Italiana del Farmaco), has an on-line monitoring dashboard for high-cost drugs, including ICIs, to ensure prescription appropriateness.

This study acknowledged several limitations beyond the retrospective design and the risk of selection bias. The dataset had not been designed for this analysis therefore we did not have information about possible treatment lines following ICIs, further hospice referrals or alternative treatment choices at ICIs initiation including clinical trials, nor about formal assessment of life expectancy at ICIs initiation. Additionally, we were unable to perform a detailed cost/benefit analysis and we did not report the irAEs incidence among the study population.

## Conclusion

Our results confirmed a trend toward increasing ICIs prescription in frail patients during the late stages of life, particularly as compared to that reported for standard chemotherapy, with questionable efficacy. Patients who received ICIs within 30 days of death and patients who started them within 3 months/30 days of death had most of the hallmarks of frailty, including a poor PS and a high burden of disease. Caution should be exercised when evaluating an considering ICIs treatment for these patients, in order to ensure an appropriate ICIs administration.

## Supplementary Information

**Additional file 1:** Table S1

## Data Availability

The datasets used during the present study are available from the corresponding author upon reasonable request.
