# Supplementary material for: PD-1/PD-L1 checkpoint inhibitors during late stages of life: an ad-hoc analysis from a large multicenter cohort
Source: J Transl Med. 2021 Jun 24;19:270. doi: 10.1186/s12967-021-02937-9 (PMC8223272; doi:10.1186/s12967-021-02937-9)
Supplement: Supplementary file 1 — Additional file 1: Table S1 [file 12967_2021_2937_MOESM1_ESM.docx]

| **Institution** | **Department** |
| --- | --- |
| St. Salvatore Hospital, University of L’Aquila, L’Aquila | Medical Oncology Department |
| University Hospital of Parma, Parma | Medical Oncology Department |
| SS Annunziata Hospital, Chieti | Medical Oncology Department |
| IRCCS Ospedale Sacro Cuore Don Calabria, Negrar | Medical Oncology Department |
| University of Bari, Department of biomedical sciences and human oncology | Medical Oncology Department |
| A.O. Papardo & Department of Human Pathology, University of Messina | Medical Oncology Department |
| IRCCS Ospedale Policlinico San Martino | Medical Oncology Department |
| S Maria Goretti Hospital, Latina | Medical Oncology Department |
| St. Andrea Hospital, Rome | Medical Oncology Department |
| Campus Bio-Medico University, Rome | Medical Oncology Department |
| Policlinico Umberto I, Rome | Medical Oncology Department |
| “UOC Oncologia Padova Sud - AULSS6 Euganea , Padova | Medical Oncology Department |
| St. Maria della Misericordia Hospital, Perugia | Medical Oncology Department |
| Hospital of Fabriano, Fabriano | Medical Oncology Department |
| Fondazione IRCCS Ca' Granda Ospedale Maggiore Policlinico, Milan | Medical Oncology Department |
| Hospital of Fermo, Fermo | Medical Oncology Department |
| Azienda Ospedaliero-Universitaria Pisana, Pisa | Medical Oncology Department |
| University Hospital of Modena | Medical Oncology Department |
| ASST Sette Laghi, Ospedale di Circolo e Fondazione Macchi, Varese | Medical Oncology Department |
| Istituto Nazionale Tumori-IRCCS Fondazione "G. Pascale", Naples, Italy | Medical Oncology Department |
| Azienda Ospedaliera S. Maria, Terni | Medical Oncology Department |
